# Intra uterine extra-amniotic versus vaginal misoprostol for termination of second trimester miscarriage: A randomized controlled trial

**Published:** 2016-10

**Authors:** Abo Bakr Abbas Mitwaly, Ahmed Mohamed Abbas, Mohamed Sayed Abdellah

**Affiliations:** *Department of Obstetrics and Gynecology, Faculty of Medicine-Assiut University, Assiut, Egypt.*

**Keywords:** *Misoprostol*, *Missed miscarriage*, *Termination of pregnancy*, *Prostaglandins*

## Abstract

**Background:**

Termination of pregnancy in the second trimester using prostaglandins has been shown to be safe and effective. Misoprostol has multiple routes of administration; oral, vaginal, buccal, rectal and sublingual.

**Objective:**

The study aims to compare the efficacy and safety of intrauterine extra-amniotic and vaginal misoprostol in a dose of 200 microgram every 4 hours for the termination of pregnancy in cases of second trimester miscarriage.

**Materials and Methods:**

A prospective randomized open labeled clinical trial included 180 women with missed miscarriage in gestational age between 13 and 24 wks. Patients were randomized to receive subsequent doses of 200 µg misoprostol every 4 hrs either intra uterine extra-amniotic by Foley catheter or vaginally administered. Randomization was completed using a computer-generated random table. The primary outcome of this study was the mean duration from the initial misoprostol dose until complete fetal expulsion (induction-expulsion interval).

**Results:**

The mean gestational age was 17.74 wks. The mean time to complete miscarriage in the intra uterine extra-amniotic group was 5.27 hrs, which was significantly lower than the vaginal group (9.92 hrs, p=0.001). Side effects were more common in vaginal group.

**Conclusion:**

Intra uterine extra-amniotic misoprostol with a dose of 200 µg every 4 hrs appears to be more effective and safer than vaginal misoprostol in induction of second trimester miscarriage.

## Introduction

The majority of second-trimester pregnancy termination performed in the United States is done surgically by dilation and evacuation (D&E) (1). The frequency of miscarriage induction increases as gestational age advances. Induction of miscarriage is considered as the primary method of termination in cases of late second trimester or early third trimester detected fetal abnormalities. However, in many countries, induction is considered as the primary method of termination all over the second trimester (2).

Termination is indicated in cases such as intrauterine fetal death, persistent heart disease after cardiac decomposition, and advanced hypertensive vascular disease (3). A lot of mechanical and pharmacological methods are listed in the literature for miscarriage induction. The safety and efficacy of each method are the main factors affecting the choice of which method of induction used. Balloon catheters exert its effect mechanically through direct pressure on the internal os of the cervix, overstretching the lower uterine segment and indirectly increasing localized prostaglandin and cytokine release (4). Medical termination offers a high possibility for improving miscarriage access and safety, due to its simplicity in comparison with surgical termination (5).

Prostaglandins (PGs) give two advantages; promoting cervical ripening and increasing myometrial contractility. Misoprostol is a synthetic analog of PGE1, which was initially licensed for the prevention and treatment of peptic ulcers. It has been used to induce miscarriage through its various routes of administration (6). One of its advantages over other drugs is that it has multiple routes of administration (oral, vaginal, buccal, rectal or sublingual) (7). However the ideal dosage and route of misoprostol still remains to be determined, with more than thirty different dosage regimens described in the literature for its use in obstetrics (8).

Termination of pregnancy in the second trimester using misoprostol has been shown to be safe and effective, with a success rate of up to 90% (9). The frequency of side effects is mainly related to the used dose and the route of administration. Most of them are gastrointestinal including nausea, vomiting, intestinal cramping, and diarrhea. Occurrence of side effects increase if higher doses used or as a result of the cumulative dose of misoprostol (10). Previous study reports that the use of titrated oral misoprostol solution could be as effective and safe for induction of labour as vaginal misoprostol (11). 

To our knowledge, there is no trials used misoprostol saline dissolute solution via intrauterine extra-amniotic route for termination of second trimester gestation. The current study aims to compare the efficacy and safety of intrauterine extra-amniotic and vaginal misoprostol in a dose of 200 μg every 4 hrs for medical termination of second trimester miscarriage.

## Materials and methods

We conducted a prospective, randomized, open labeled, controlled trial (Clinical Trials. Gov; NCT02669420) in Assiut Women’s Health Hospital, Egypt between the 1^st^ of February and the 1^st^ of December 2015. The Assiut University Medical Ethical Review Board approved the study (IRB approval # 00009898). The participants were recruited from the Labor Ward. 

**Eligible participants**

All women presented with second trimester miscarriage to our hospital were invited to participate. Informed consent was obtained for participation after discussing the nature of the study including the possible side effects of misoprostol. The recruited women were entered the screening phase of the study. This phase included history taking (including age, parity and gestational age) and clinical examination included BMI, and vital signs assessment. 

We included in our study pregnant women (13-24 wks), with intrauterine fetal death confirmed by ultrasound examination via 2 different sonographers. Exclusion criteria included women with a previous caesarean section, low lying placenta, known allergy to misoprostol, premature preterm rupture of the membranes, history of recent asthma, a known coagulation disorder and history of chronic adrenal failure. Women who refused to participate in the study were also excluded.

**Randomization**

A statistician prepared a computer generated random table and placed the allocation data in serially numbered sealed envelopes. Each envelope had a card noting the intervention type inside. The envelopes opened only by the clinician according to the order of attendance of women. After acceptance of eligible women to participate in the study, we assigned them randomly in a 1:1 ratio to receive either Intra uterine extra-amniotic or vaginal misoprostol. Allocation unchanged after opening the closed envelopes. 

**Intervention**

All participants were allocated to one of two groups; the group A (Intra uterine extra-amniotic misoprostol group) received one tablet of misoprostol (Misotac, Sigma, Egypt) containing 200 µg in saline dissolute solution through intra uterine Foley's catheter inserted for every patient every 4 hrs. Group B (vaginal misoprostol group) received one tablet of misoprostol vaginally every 4 hrs. In group A; Foley's catheter (16 French) introduced transcervically with direct visualization by use of a vaginal speculum under complete sterile conditions in the operative theater. Cleaning of the cervix with an aseptic solution (chlorhexidine) was done. After passing the internal os, the catheter balloon was inflated with 30 ml of sterile water, and the external end of the catheter was taped to the thigh.

Infusion set was fixed to the Foley's catheter at opening of its drainage side after cutting part of this opening to allow tight fixation of the infusion set. Then, an infusion of 500 cc of saline loaded by 500 mg misoprostol (2 and half tablets of misoprostol solved in this 500 cc of saline) and flow by rate of 20 drops/min equal to 2 mg of misoprostol extra-amniotically per minute, as each milliliter of normal saline contain now one microgram of misoprostol. 

This means that the dose of 20 drops/min contain only 2 mg of misoprostol solution and no incremental increase in the dose by increasing the drops. When the catheter was expelled from the vagina spontaneously, the patient was examined and oxytocin infusion started after expulsion of the fetus and the placenta. Prophylactic broad spectrum antibiotic, third generation cephalosporin intravenously, was given in a regular base every 12 hrs during the procedure. Group B received a dose of moistened, by distilled water, misoprostol (200 μg) every 4 hrs vaginally, a tablet was put into the posterior fornix. The same antibiotic was given every 12 hrs during the procedure.

**Study outcomes**

The primary outcome of this study was the mean duration from the initial use of misoprostol till fetal expulsion completed (induction-expulsion interval). The secondary outcomes included the dose of used misoprostol, the need for analgesics, need for surgical evacuation in cases of retained placenta, and lastly; occurrence of side effects.

**Follow up**

Monitoring of blood pressure, temperature, side effects, and bleeding were occurred every 4 hrs after initiation of misoprostol. Oral or parenteral analgesia was provided if requested. Complete procedure is considered when the products of conception were expelled completely (including the placenta) within 24 hrs of the starting dose of misoprostol so, no further interventions were needed. If fetal expulsion did not occur within 24 hrs from initiation of misoprostol, the induction was considered a failure and the woman was offered standard evacuation. However, if the fetus was expelled but the placenta remained in the uterus after an additional 30 min, the woman could be given an oxytocin intravenous infusion to help placental expulsion. If placental expulsion still did not occur or there was heavy bleeding, the investigator was instructed to remove remaining products surgically. All women were discharged within 48 hrs.

**Sample size**

Sample size was calculated using the Open Epi software program, version 2.3.1. Previous multicentric study in 2009 considered the rate of successful miscarriages after 48 hours of receiving vaginal misoprostol as 96%. Using two sided chi-square (^2^) test with α of 0.05, a total sample size of at least 162 women in both groups (n=81) using 80% power would be necessary to detect a 30% difference in both groups [OR:5.8]. We expected a 10% dropout rate; therefore 180 women were recruited to the study.

**Statistical analysis**

The data were collected and analyzed using the Statistical Package for Social Science (SPSS Inc., Chicago, version 21). The demographic data were compared between treatment groups. The outcome variables were calculated using a student t-test to compare continuous variables. For dichotomous variables, ^2^ was used to estimate the significance value. For analysis, p<0.05 was considered to be significant.

## Results

Out of 204 eligible patients presented to our hospital, 180 consented to participate. Twenty women didn't meet the inclusion criteria and 4 women were not willing to share in the study. Consenting women were randomized into two groups: Intra uterine extra-amniotic misoprostol group and vaginal misoprostol group (Figure 1). Table I show that both groups were comparable in the baseline characteristics. There was no significant difference in vital signs between both groups. 

The results for primary and secondary outcomes are presented in table II. There are no cases of failure in both groups. The mean duration of induction-expulsion interval in intra uterine extra amniotic group was significantly shorter than vaginal group (5.11±2.66 vs. 9.92±3.12 hrs respectively; p=0.001). The mean dose of received misoprostol (µg) was significantly lower in the intra uterine extra-amniotic group (263±219.7 vs. 508±198.2 µg respectively; p=0.000). There was significant difference in the cases with retained tissue needed surgical evacuation between the two groups (14% vs. 5.5%, p=0.005).

There was also significant difference in the requirement for analgesics between the two groups (33% vs. 11%, p=0.005). All women were observed and asked about side effects throughout induction of abortion. Nausea and vomiting in intra uterine extra amniotic group were significantly less than vaginal group (Table III).

**Table I:** Baseline characteristics of patients at inclusion in both groups

**Variables**	**Intra uterine extra amniotic group**	**Vaginal group**	**p-value**
Age (yrs)	32.17 ± 6.07	31.54 ± 5.23	0. 219
Parity	2.47 ± 1.61	2.00 ± 1.44	0.426
BMI (Kg/m^2^)	26.35 ±3.6	26.11±1.2	0.321
Gestational age (wks)	17.87 ± 2.79	17.60 ± 2.69	0.515
Pulse (beats/min)	79.70 ± 6.14	79.48 ± 5.93	0.805
Systolic BP (mmHg)	119.33 ± 15.92	119.44 ± 14.01	0.960
Diastolic BP (mmHg)	69.02 ± 15.13	69.40 ± 9.48	0.841

Data are presented as mean±SD (n=90). Student’s t-test was used to determine p-value between both groups

BMI; body mass index

BP; blood pressure

SD; standard deviation

**Table II:** The study outcomes in both groups

**Outcomes **	**Intra uterine extra amniotic group**	**Vaginal group**	**p-value** a
Induction-expulsion time interval (hrs)*	5.11 ± 2.66	9.92 ± 3.12	0.001*
Mean dose of misoprostol until expulsion (µg)*	263 ± 219.7	508 ± 198.2	0.000*
Number of women needs surgical evacuation, n (%)**	5 (5.5%)	13 (14%)	0.005*
Number of women needs analgesics, n (%)**	10 (11%)	30 (33%)	0.005*

Student’s t-test in quantitative data and Chi-square test in qualitative data were used to determine the p-value between groups

* Data are presented as mean±SD (n=90)

** Data are presented as n (%)

a Statistical significant difference

**Table III:** Side effects associated with misoprostol in both groups

**Side effects**	**Intra uterine extra amniotic group**	**Vaginal group**	**p-value**
Nausea	2(2.2)	34 (37.8)	0.000*
Vomiting	2 (2.2)	8 (8.9)	0.005*
Diarrhea	0 (0)	2 (2.2)	---
Fever	4(4.4)	0 (0)	---
Chills	0 (0)	20 (22.2)	---

Data are presented as n (%) (n=90). ^2^ test was used to determine the p-value between groups

* Statistical significant difference

**Figure I:**
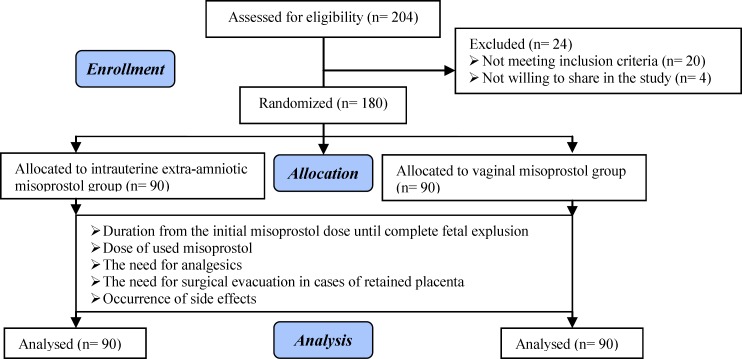
The study flowchart

## Discussion

This is the first reported RCT to investigates the effect of misoprostol saline dissolute solution via intrauterine extra-amniotic route for termination of second trimester miscarriage. Our study proved that the use of intra uterine extra-amniotic misoprostol with a dose of 200 ug every 4 hours is more effective and safer than vaginal misoprostol.

Second trimester pregnancy termination has been reported to be associated with 3-5 times higher maternal morbidity and mortality risks than first trimester termination (12). There are several lines published in literature for medical termination of second trimester miscarriage. Most of studies depend on misoprostol, a synthetic prostaglandin E1 analogue (PGE1), whether used alone or combined with mifepristone (8). Previous studies of medical induction of second trimester miscarriage using both mifepristone and misoprostol reported to have the highest efficacy and the shortest time interval (13). Misoprostol alone is a logical alternative if no available mifepristone.

Misoprostol (PGE1) is increasingly used for second trimester termination of pregnancy (14, 15). It is inexpensive, stable at room temperature and it is rapidly absorbed by sublingual, vaginal and oral routes, so it is commonly used in Egypt for induction of miscarriage in the second trimester (16, 17). Different studies were performed to evaluate misoprostol, vaginally, orally, sublingually or buccally for second-trimester termination, with different reports of success rate of pregnancy termination in 24 hrs, in 48 hrs, the time interval between induction and fetal expulsion and the side effects in all groups (8). 

The results of previous studies concluded that vaginal administration of misoprostol is associated with slower absorption lower peak plasma levels, greater exposure to the drug and greater effects on the cervix with lower rate of gastrointestinal side effects (8, 18). The main drawbacks of vaginal misoprostol use are the possible effect of vaginal bleeding and vaginal pH on the drug bioavailability when administered vaginally. To our knowledge no one used titrated misoprostol solution extra-amniotically via Foley catheter for pregnancy termination during second trimester gestation. Previous trials tested injection of PGF2α or ethacridine through the cervix into the extra-amniotic space using a Foley catheter (19). Although they are effective in inducing miscarriage, they were associated with higher rates of serious adverse events (19).

Combined use of intracervical Foley catheter, as a mechanical method for induction, and vaginal misoprostol for induction of second trimester miscarriage was assessed in recent studies with contrary results (20, 21). Toptas *et al* reported in their study that combined vaginal misoprostol with Foley catheter insertion had similar efficacy to that of vaginal misoprostol alone as regard time to abortion/birth, 14.33 and 12.08 hours, respectively (21). 

In our study, Induction-expulsion interval in intra uterine extra-amniotic group (5.11±2.66 hrs) was significantly shorter than vaginal group (9.92±3.12 hrs), this agree to study done by Rezk *et al* who reported a high success rate with shorter induction-expulsion interval when use of intracervical Foley's catheter combined with misoprostol in comparison to either misoprostol alone or Foley's catheter alone for termination of second trimester pregnancy (20). In our study the dose was repeated every 4 hours with high success rate in both groups, this was nearly similar to the results of Wong et al who found that the induction-miscarriage interval with use of vaginal prostaglandins every 3 hrs is shorter than every 6 hrs administration without any extra side effects (22).

The side effects and need of analgesia of extra-amniotic misoprostol use were comparable to that achieved by Rezk *et al* (20). In our study there were no major complications encountered and no maternal mortality. The main strengths of our study were that it was randomized study, and included big sample size for testing the use of intrauterine extra-amniotic route of misoprostol. The limitations of the study were that the tested route of misoprostol used only with missed miscarriage and the acceptability of the intervention was not assessed by questionnaire at follow-up visit. 

## Conclusion

In conclusion, intrauterine extra-amniotic misoprostol solution appears to be an effective and safe route for termination of second trimester missed miscarriage with shorter induction expulsion interval. Further larger sample size randomized clinical studies are needed to confirm our results. 

## Conflict of interest

The authors declare that they have no conflict of interest.

## Note

***Registration ID in*** ***Clinical Trials.Gov*** ***:*** ***NCT02669420***
